# Inhibition of corticotropin releasing factor expression in the central nucleus of the amygdala attenuates stress-induced behavioral and endocrine responses

**DOI:** 10.3389/fnins.2013.00195

**Published:** 2013-10-29

**Authors:** Leah B. Callahan, Kristi E. Tschetter, Patrick J. Ronan

**Affiliations:** ^1^Avera Research Institute, Avera McKennan Hospital and University Health CenterSioux Falls, SD, USA; ^2^Neuroscience Group, Division of Basic Biomedical Sciences, University of South Dakota Sanford School of MedicineVermillion, SD, USA; ^3^Department of Psychiatry, University of South Dakota Sanford School of MedicineSioux Falls, SD, USA; ^4^Research Service, Sioux Falls VA Health Care SystemSioux Falls, SD, USA

**Keywords:** CRF, RNA interference, CeA, corticosterone, stress, anxiety, knockdown

## Abstract

Corticotropin releasing factor (CRF) is a primary mediator of endocrine, autonomic and behavioral stress responses. Studies in both humans and animal models have implicated CRF in a wide-variety of psychiatric conditions including anxiety disorders such as post-traumatic stress disorder (PTSD), depression, sleep disorders and addiction among others. The central nucleus of the amygdala (CeA), a key limbic structure with one of the highest concentrations of CRF-producing cells outside of the hypothalamus, has been implicated in anxiety-like behavior and a number of stress-induced disorders. This study investigated the specific role of CRF in the CeA on both endocrine and behavioral responses to stress. We used RNA Interference (RNAi) techniques to locally and specifically knockdown CRF expression in CeA. Behavior was assessed using the elevated plus maze (EPM) and open field test (OF). Knocking down CRF expression in the CeA had no significant effect on measures of anxiety-like behavior in these tests. However, it did have an effect on grooming behavior, a CRF-induced behavior. Prior exposure to a stressor sensitized an amygdalar CRF effect on stress-induced HPA activation. In these stress-challenged animals silencing CRF in the CeA significantly attenuated corticosterone responses to a subsequent behavioral stressor. Thus, it appears that while CRF projecting from the CeA does not play a significant role in the expression stress-induced anxiety-like behaviors on the EPM and OF it does play a critical role in stress-induced HPA activation.

## Introduction

Corticotropin releasing factor (CRF), also known as corticotropin releasing hormone or corticoliberin, is a 41 amino acid peptide (Vale et al., 1981) that plays an essential role in stress adaptation and coping. It regulates a wide range of both acute and chronic neuroendocrine, autonomic, and behavioral responses (Koob and Bloom, 1985; Dunn and Berridge, 1990; Heinrichs et al., 1995; Lehnert et al., 1998; Ronan and Summers, 2011). Dysregulation of CRF signaling may be a common molecular pathway for the myriad “stress-related” disorders. Measures of hyperactivity of the CRF system have been consistently implicated in the pathophysiology/etiology of a number of psychiatric conditions including anxiety disorders such as PTSD as well as depression and addiction (for reviews, see Arborelius et al., 1999; Bale and Vale, 2004; Ronan and Summers, 2011). Understanding the complexities of CRF regulated signaling and behaviors is essential to understanding these psychiatric disorders.

Many studies have revealed a role for CRF and CRF_1_ receptors in a range of stress responses. In animal models CRF (icv) induces anxiety-like behavior and depressive symptoms, such as anhedonia, decreased appetite, reduced slow wave sleep, psychomotor alterations and reduced libido (Keck, 2006; Binder and Nemeroff, 2010). The anxiogenic character of endogenous CRF receptor ligands is verified by consistent anxiolytic effects of peptide and non-peptide CRF_1_ antagonists (Zorrilla and Koob, 2004; Holsboer and Ising, 2008), and by reduced anxiety-like behavior in mice with a conditional knockout of limbic brain CRF_1_ receptors (Muller et al., 2003; Nguyen et al., 2006). Thus, during stress CRF_1_ receptor signaling produces anxious behavior (Coste et al., 2001; Risbrough et al., 2003, 2004, 2009; Bale and Vale, 2004; Heinrichs and Koob, 2004). Further results show decreased responsiveness to stressful stimuli in constitutive transgenic CRF_1_ receptor knockout mice (Smith et al., 1998; Timpl et al., 1998; Contarino et al., 1999). Together these findings suggested that the CRF system would play an important role in depression and anxiety disorders.

The central nucleus of the amygdala (CeA) is a key component of an extended brain circuitry involved in stress responses and anxiety-like behaviors and is an important structure for CRF-mediated responses as it contains one of the highest concentrations of CRF immunoreactive neurons outside of the PVN (Cummings et al., 1983; Swanson et al., 1983; Cassell and Gray, 1989; Shimada et al., 1989). Numerous studies have shown that a wide variety of stressors and anxiogenic stimuli activate neurons of the CeA as measured by increased expression of the early immediate gene c-fos (Hayward et al., 1990; Campeau et al., 1991; Honkaniemi et al., 1992; Funk et al., 2003; Asok et al., 2013) supporting the notion that stressors act, at least in part, through the CeA. A vast neuroimaging literature has linked hyperresponsivity of the amygdala to a range of anxiety and stress-induced disorders such as PTSD (Liberzon and Sripada, 2008; Hughes and Shin, 2011), addiction (Crunelle et al., 2012; Mihov and Hurlemann, 2012; Goldman et al., 2013) and depression (Bellani et al., 2011a,b). The CeA is the primary output of the amygdala. Fibers from dense pyramidal CRF neurons located primarily in the lateral part of the CeA project to other key regions including lateral hypothalamus, lateral BNST, mesencephalic reticular formation, locus ceruleus, raphé, ventral tegmental area, dorsal and ventral parabrachial nuclei, mesencephalic nucleus of the trigeminal nerve, core and shell ventromedial hypothalamus, ventral subiculum, corticomedial amygdala (Cummings et al., 1983; Sakanaka et al., 1986; Rodaros et al., 2007). Evidence suggests that CeA CRF neurons play a role in HPA axis activation. Few fibers from the CeA innervate the PVN directly (Sawchenko et al., 1993) but fibers do heavily innervate PVN projecting structures such as the BNST and very likely regulate the release of CRF from PVN to the pituitary where it is the primary secretagogue of ACTH causing HPA axis activation during stress (Choi et al., 2008).

Studies have suggested that CRF neurons in the CeA specifically mediate a range of behavioral and endocrine responses to stressors (Heinrichs et al., 1992; Heilig et al., 1994; Gray and Bingaman, 1996). Stressors, both physical and psychological, cause a rapid increase (within 1–3 h) in the expression of CRF mRNA (Mamalaki et al., 1992; Kalin et al., 1994; Hsu et al., 1998; Makino et al., 1999) and peptide (Makino et al., 1999). Administration of CRF (icv) elicits anxiety-like behaviors that are attenuated by infusion of CRF antagonists into the CeA (Heinrichs et al., 1992; Rassnick et al., 1993; Swiergiel et al., 1993). This suggests that CRF neurons in the CeA are involved in these behaviors since the majority of CRF in the CeA likely arises from local circuits (Merlo-Pich et al., 1995). Studies utilizing different animal models including rats, cats, and rabbits have shown that electrical stimulation of the CeA causes autonomic and anxiety-like responses mimicking those elicited by stress that are identical to responses caused by icv CRF administration (Hilton and Zbrozyna, 1963; Mogenson and Calaresu, 1973; Stock et al., 1978). Microdialysis studies have demonstrated local CRF release in the CeA during restraint stress (Merlo-Pich et al., 1995). Increased release of CRF in the CeA is also implicated as a potential mechanism underlying the anxiety associated with withdrawal in drug and alcohol abuse (Menzaghi et al., 1994; Merlo-Pich et al., 1995; Richter et al., 1995, 2000; Richter and Weiss, 1999). Lesions of the amygdala block fear-conditioned startle responses (Hitchcock and Davis, 1991). Bilateral lesions of the CeA in rhesus monkeys reduced fear-related behavior and freezing when exposed to an aversive stimulus and decreased levels of cerebral spinal fluid CRF and plasma ACTH in response to the stressor. This study showed that the CeA is involved in regulating hypothalamic CRF activity, peripheral endocrine responses and behavioral responses to fear-eliciting stimuli (Kalin et al., 2004). More recently, studies on adult mice showed that site specific manipulation of CeA CRF expression through lentiviral-based systems affected behavioral and endocrine systems (Regev et al., 2011, 2012; Flandreau et al., 2012) with sometimes contradictory results. In contrast to the wealth of evidence pointing to a role for CRF in the CeA in stress-induced anxiety-like behavior, long-term (4 month) viral-mediated overexpression of CRF in the CeA actually reduced anxiety-like behaviors (Regev et al., 2011) whereas long-term knockdown leads to decreases in anxiety-like behavior and cortisol responses to acute stressors (Regev et al., 2012).

Though a role of the CeA in stress and anxiety responses has been extensively characterized, the specific contribution of CRF neurons in the CeA to these responses has not. There are many other neurotransmitter systems in the CeA that could be playing a role including, among others, NPY and neurotensin. It has been estimated that up to 90% of neurotensin labeled cells in the CeA are also immunoreactive for CRF (Shimada et al., 1989). Also, most studies implicating the CeA in stress responsiveness have relied on stimulation or lesions; both of which could have non-specific effects. For example, fibers projecting from the basolateral amygdala to the BNST pass directly through the CeA. Some have speculated that electrolytic lesions of this pathway account for effects of what had been ascribed to CeA lesions (Davis et al., 2010).

To further clarify the role of CRF in the CeA on anxiety-like behavioral and corticosterone endocrine responses to stressors we silenced expression of CRF in the CeA of adult rats using RNA interference (RNAi). Our goal was to achieve a rapid localized knockdown of CRF to limit possible compensatory upregulation of other stress responsive systems that could mask the true role of amygdalar CRF in these responses. We hypothesized that unconditioned anxiety-like behaviors and corticosterone responses to stressors are mediated by CRF in the CeA and knocking down CRF expression would be sufficient to attenuate these responses.

## Materials and methods

### Animals

Adult male Sprague-Dawley rats (Charles River; 225–250 g) were purchased from Charles River Breeders (CD-IGS Strain, Wilmington, MA). Rats were housed 2 to a cage (24′ × 10′), given free access to food and water and maintained on a 12:12 Light: dark schedule with lights on at 7:00 am. Rats were acclimated for at least 1 week before any procedures were carried out. All animal procedures were carried out under an approved IACUC protocol from the University of South Dakota in accordance with all applicable rules and regulations in an AALAC approved facility at the Sioux Falls VA Medical Center.

### RNA interference

Short 21-mer double-stranded RNA oligonucleotides with TT overhangs (siRNA) were synthesized (IDT DNA, Coralville, IA) targeting the open reading frame of rat CRF mRNA (accession number NM_031019). Multiple candidate 21-mer targets of the CRF coding region of the CRF precursor gene were chosen using the web-based SDS Program (siRNA Design Software; http://i.cs.hku.hk/~sirna/software/sirna.php). This software tool makes use of multiple existing design tools to output a set of ranked candidate targets. These targets were further filtered based on RNA secondary structure using the online bioinformatics site mfold Web Server (Zuker, 2003; http://mfold.rna.albany.edu/?q=mfold/RNA-Folding-Form). The selected target sequence has ~50 G-C content and is located within one exon (mRNA nucleotides 697–716). A BLAST search confirmed uniqueness of sequence.

#### Duplex sequences

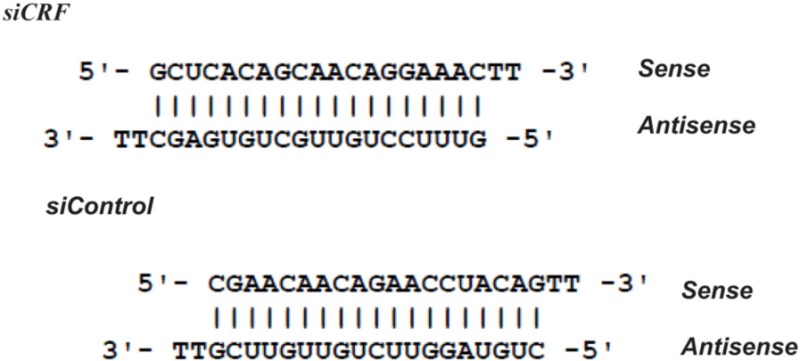



Sense and antisense RNA strands were synthesized with 5′ tt overhangs (IDT DNA, Coralville, IA). The siRNA oligonucleotides were suspended in RNase-Free Duplex Buffer (IDT DNA, Coralville, IA). GeneSilencer siRNA transfection reagent (Gene Therapy Systems San Diego, CA) was mixed (1:1 ratio) with the diluted siRNA oligonucleotides for a final siRNA concentration of 3.3 μg/μl. The mixture was allowed to incubate at room temperature (RT) for 5 min before it was loaded into a sterilized RNase-free syringe.

### Surgery

Rats were anesthetized with isoflurane (2–2.5%; VetEquip Inhalation Anesthesia System, Pleasanton, CA). Scalps were shaved and rats were placed in a stereotactic apparatus (Kopf model #900, Tujunga, CA) with the incisor bar set to −3.3 to attain a flat skull position. Eye drops (LiquiTears™) were gently applied throughout the surgery to prevent irritation and drying. A Betadine scrub solution (provodone-iodine 7.5%) was applied to the surgical area three times with fresh, sterile cotton swabs; each time removed using sterile saline. A longitudinal incision was made in the scalp and sterile tissue clamps were applied to expose the skull. Fascia was scored and gently scraped with a scalpel. A dilute hydrogen peroxide solution was applied to assist with stopping any bleeding and it makes Bregma more visible. Stereotactic coordinates were determined for the CeA (AP −2.1; ML ± 4.25; DV −8.4; Paxinos and Watson, 1997) and holes drilled through the skull with a sterile 0.7 mm burr bit using a precision drill (Foredom) attached to a stereotactic arm, being careful to leave dura intact. With the aid of a surgical stereomicroscope, dura and any remaining bone disc were very carefully removed using a sterile 25-gauge needle. Sterilized 10 μl syringes were filled with either an siRNA duplex targeting CRF mRNA (siCRF) or scrambled sequence (siControl) diluted with transfection reagent as described above. Syringes were placed in a microinjector unit attached to the stereotactic frame (Model 5000/5001, David Kopf Instruments, Tujunga, CA) and the syringe tip zeroed at the brain surface. The syringe was then slowly (30–45 s) lowered to the correct depth. The injections were made with a slow, steady rate of injection (1.5 μl/15 min). This procedure was repeated on the contralateral side of the brain using the same RNAi duplex. For the dose response testing, the contralateral side of the brain received the scrambled RNAi duplex. Bone wax was applied to the drilled holes and the incision closed with staples (9 mm EZ Clips™). Triple antibiotic cream was applied to the wound and the rat was placed in a cage with an absorbable lining and a 125-Watt infrared heat lamp suspended over one end. Rats were closely monitored until fully ambulatory. Rats were given buprenorphine (0.01 mg/kg SQ) and returned to their housing unit where they were individually housed until experimental procedures.

### Dose response and time course of the siRNA

A preliminary dose and time course experiment was run to determine the lowest effective dose that could be used for behavioral experiments. In order to evaluate the role of CRF in the CeA on endocrine and behavioral responses to stressors and minimize the potential for compensatory upregulation of other stress systems we wanted to find the most effective dose with the shortest latency to loss of peptide. Rats (*n* = 2/group) received unilateral stereotactic injections of three different concentrations of siRNA oligonucleotides were tested: 6.6 μg/μl, 3.3 μg/μl, 1.7 μg/μl. One side received siRNA oligonucleotides targeting CRF mRNA (siCRF) and the contralateral side received a scrambled sequence (siControl), into the CeA. Three different time points were tested: 24, 48, and 72 h post-injection. Rats were killed and brains removed for histological verification of both injection site and quantification of CRF knockdown.

### Verification of CRF knockdown

#### Immunohistochemistry

Immunostaining for CRF on brain sections containing the CeA was performed. Rats were deeply anesthetized with isoflurane and perfused transcardially with 300 ml ice-cold saline (0.9%) with heparin (200 units/l) followed by 300 ml fresh 4% paraformaldehyde in 1X PBS using a gravity perfusion system. Brains were removed, post-fixed in 4% paraformaldehyde overnight (24 h) at 4°C then cryoprotected in 20% glycerol in 1X PBS before being immersed in OCT compound (Tissue-Tek, Sakura) frozen surrounded by powdered dry ice. Brains were sectioned at 35 μm on a cryostat microtome (chamber −25°C, specimen −21°C; Leica CM 1900, Leica Microsystems; Wetzlar, Germany) and stored at 4°C in 1X PBS with 0.01% NaAzide (Sigma, St. Louis, MO). Sections containing the CeA were chosen with reference to an atlas (Paxinos and Watson, 1997) washed twice for 5 min each in 1X PBS then placed in blocking buffer (3% Normal Goat Serum, 0.25% Triton X-100 in 1X PBS) for 60–90 min. Primary antibodies were diluted with blocking buffer (CRF 1:1000, rabbit anti-rat polyclonal cat# T-4037; Peninsula Labs, Belmont, CA; NeuN 1:500, mouse anti- rat monoclonal cat# MAB377; Millipore, Billerica, MA). Sections were incubated overnight (~18 h, free floating) at RT with gentle agitation. The next day sections were rinsed 3 × 3 min in 1X PBS then incubated with fluorescently labeled secondary antibody (Cy2 or Cy3 Goat anti-rabbit or goat anti-mouse IgG; Jackson ImmunoResearch, West Grove, PA) diluted 1:200 in 1X PBS and incubated at RT in darkness with gentle agitation for 2–4 h. Sections were washed in 1X PBS three times for 3 min, mounted on poly-l-lysine coated and dried for 2 h. Slides were then washed in 1X PBS followed by an ethanol dehydration series (50, 75, 90, 100, 100% for 10 s each) and the xylene substitute Citrisolv (Fisher) 2 × 3 min before being coverslipped with DPX mountant (Fluka).

#### Microscopy

A Zeiss Axioskop microscope equipped with an Axiocam color camera was used to acquire digital images of CRF immunofluorescence in CeA using a 20x objective from 3–4 tissue sections from each animal. All images were acquired using identical parameters. Acquisition settings were established to prevent overexposure using native CRF immunoflourescence from homecage rats. Images from coded slides were analyzed using Adobe Photoshop 7.0 (Adobe, Mountain View, CA). Using a selection tool the CeA was manually selected and average luminosity (intensity) level ± SD of the pixels calculated using the histogram function. All values were normalized for differences in background fluorescence by selecting a standardized unlabeled area adjacent to the CeA in the same section. Corrected luminosity values were calculated by subtracting this background luminosity from luminosity in the CeA. These values were averaged for each animal.

### Behavioral testing

Separate cohorts of rats for open field (OF) and elevated plus maze (EPM) testing were randomized into four groups (*n* = 9–12/group): (1) No Stress-siControl, (2) No Stress-siCRF, (3) Stress-siControl, and (4) Stress-siCRF. Separate cohorts were used to enable us to decipher the effects of prior stress on these behaviors. We were very cautious to control the amount of prior stress the animals had since that was one of our experimental conditions. Animals in the Stress groups were restrained 24 h prior to behavioral testing. Restraint stress was administered for 1 h in commercial acrylic restraint tube with extra ventilation holes. All testing was digitally recorded and analysis performed with Ethovison XP software (Noldus Information Technologies, Netherlands). Immediately following behavioral testing rats were killed and brains removed for histological verification of both injection site and knockdown of the CRF peptide in CeA.

#### Elevated plus maze

Rats were placed in the center of the EPM and behaviors recorded with a camera mounted on the ceiling for 5 min. Duration and frequency of open vs. closed arm entries along with total locomotion were quantified using Ethovision software. Other measures of exploratory and anxiety-like behaviors including head peaking (defined as breaking the plane of the entrance to open arm with their body remaining in the center), rearing and grooming were scored by two blinded observers.

#### Open field

Rats were placed in the center of the OF (oval field, 61.0 × 91.4 cm) and allowed to stay in the OF for 10 min. Frequency and duration of entries in the center zone along with rearing and locomotion were measured.

### Plasma corticosterone

Blood samples (~25–50 μl) were quickly collected (<30 s) from the tail vein of rats using heparinized capillary tubes after both restraint stress and behavioral testing as well as from home cage control rats. Blood was centrifuged, plasma collected and stored at −20°C until assay. Corticosterone concentrations were determined by ELISA (R&D Systems, Minneapolis, MN).

### Statistics

Corrected luminosity values of siCRF and siControl rats were compared using an unpaired *t*-test. Data for corticosterone and behaviors as dependent variables were analyzed by two-way ANOVA (prior stress exposure X siRNA treatment) using SigmaStat 3.0 (Systat Inc.). Pairwise comparisons were performed using the Bonferroni *post-hoc* test. Significance level was set at α = 0.05.

## Results

### Effectivenes of siRNA knockdown of CRF

**Figure 1:**
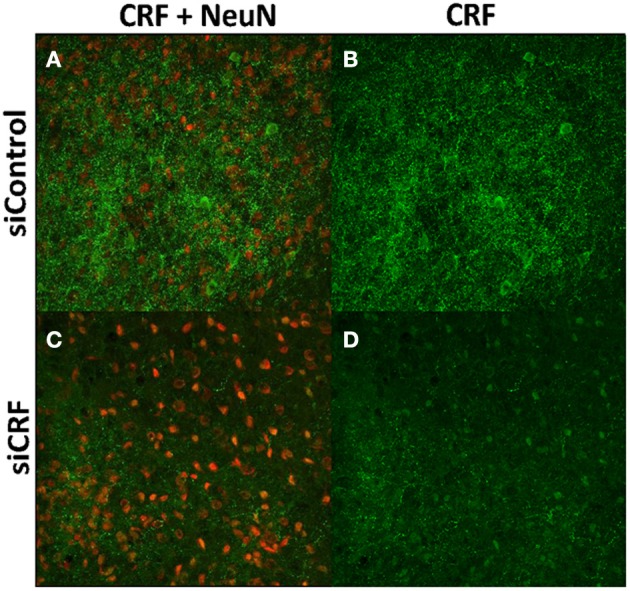
**Images (40X, Z-Scan) showing immunoreactivity for CRF (green) and NeuN (red) in the CeA of siControl injected (A,B) and siCRF injected CeA (C,D; 1.5 μl, 3.3 μg, 48 h post-injection).** Immunoreactivity for CRF is robust in CeA of siControl injected while siCRF causes a substantial decrease in CRF signal with no effect on number of neurons.

**Figure 2:**
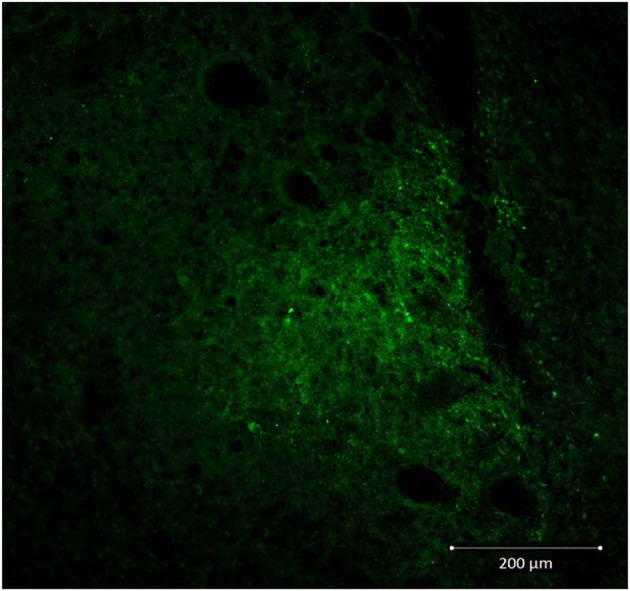
**Representative image (20X) showing injection needle tract in CeA of a siControl injected animal.** CRF neurons are labeled in green.

**Figure 3:**
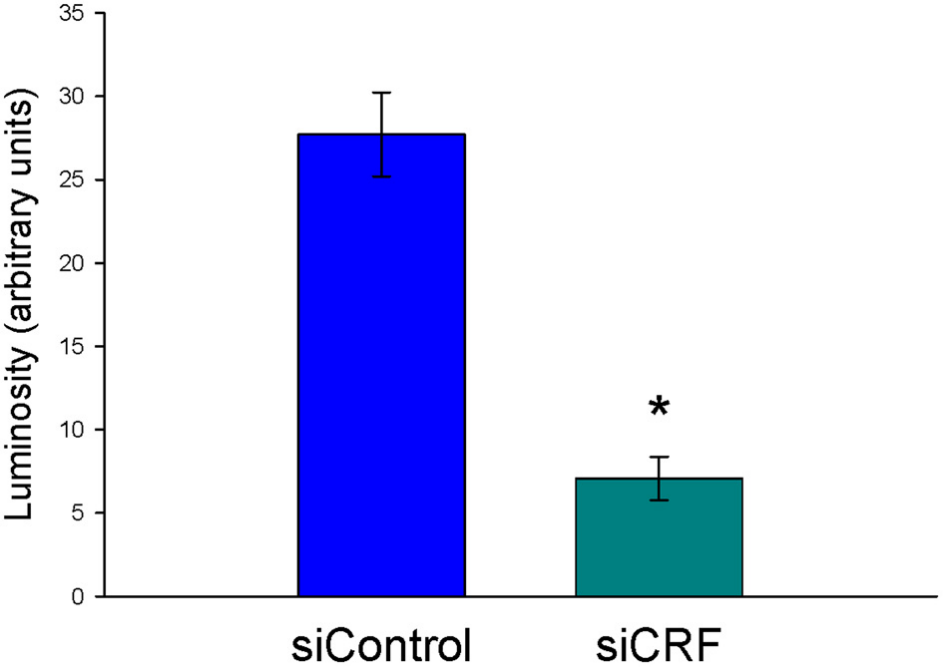
**Corrected luminosity (arbitrary units) of CRF immunofluorescence within the CeA of control (scrRNA) and siRNA injected rats.** 1.5 μl injections (3.3 μg siRNA) of either scrambled siRNA control or siRNA targeting CRF mRNA (^*^p = 0.00001).

Decreased expression of CRF peptide with siRNA treatment was evident across a wide range of doses (6.6 μg/μl, 3.3 μg/μl, 1.7 μg/μl) and time points (24, 48, 72 h). Based on the results from this preliminary investigation we chose to use 3.3 μg/μl in these sets of experiments because it was the lowest consistently effective dose at 48 h. Immunohistochemical labeling of CRF and the neuron specific marker NeuN in the CeA shows an almost complete siRNA-induced silencing of CRF expression without any loss of cells (Figure 1). Figure 2 shows a representative image of the mechanical lesion caused by the injector needle in the CeA. Corrected luminosity values of CRF immunofluorescence within the CeA of control (siControl) and siCRF injected rats (Figure 3; *p* < 0.00001).

### Plasma corticosterone

**Figure 4:**
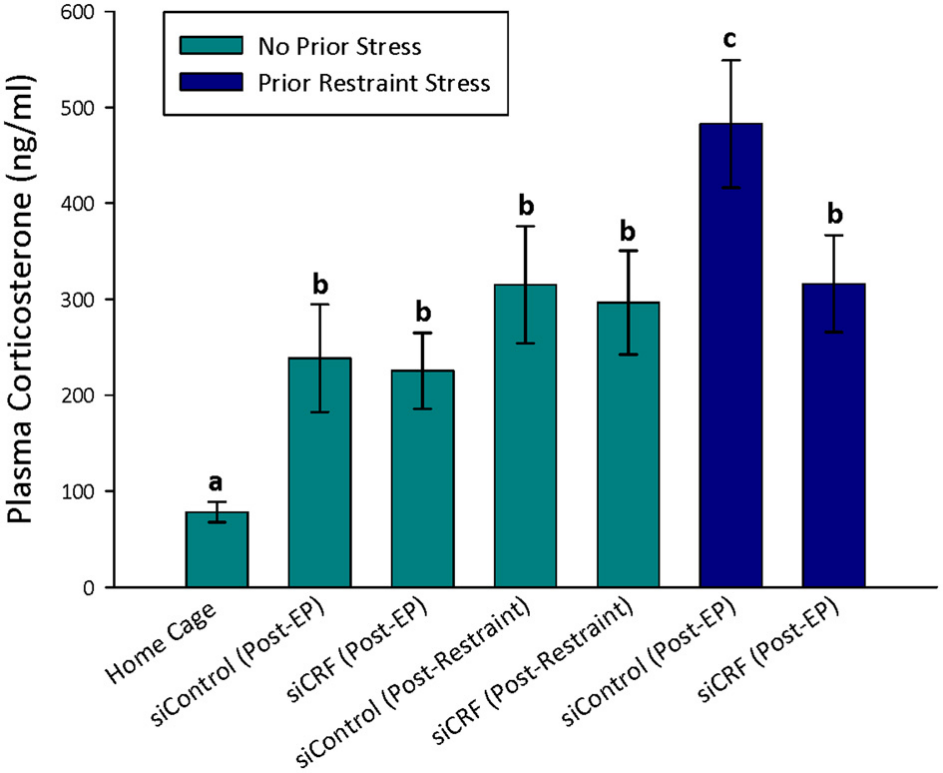
**Plasma corticosterone concentrations after restraint stress and EP testing in siContol and siCRF rats.** Restraint stress and EPM induced a robust increase in corticosterone in both control and treatment rats (*p* < 0.001). The highest concentrations of plasma corticosterone were found after EPM testing in rats that had received restraint stress 24 h prior. This restraint stress sensitization of corticosterone response to a subsequent stressor was significantly attenuated by siRNA treatment (*p* < 0.05).

**Figure 5:**
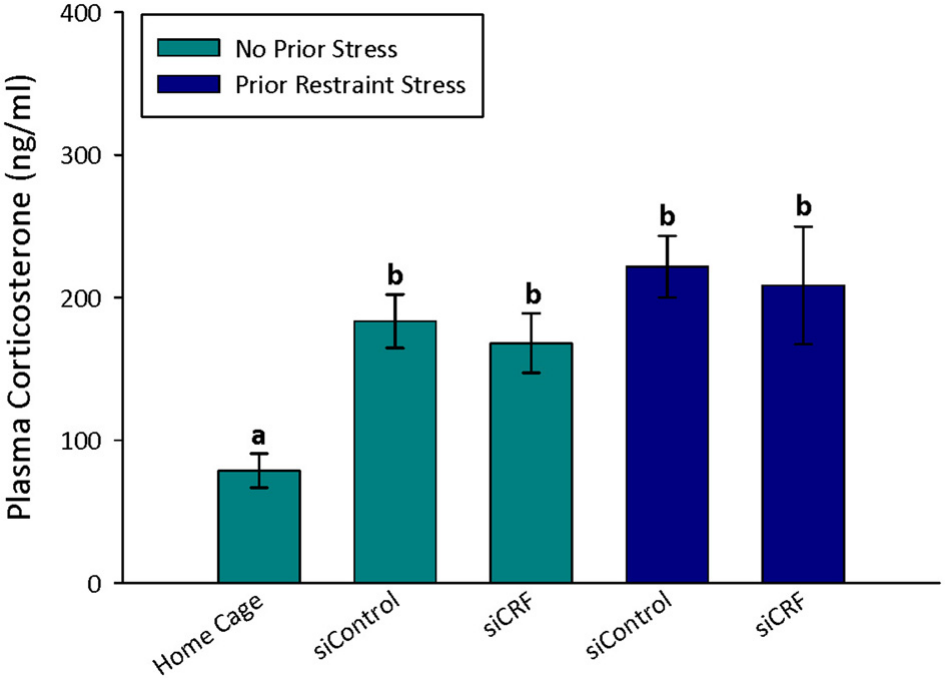
**Plasma corticosterone values after open field.** Treatment with siCRF and prior restraint had no significant effect on the increase in plasma corticosterone induced by OF testing (*p* < 0.001).

As would be expected, home cage control rats had significantly lower concentrations of plasma corticosterone than rats that were either restrained [*F*_(6, 74)_ = 12.82; *p* < 0.00]) or after behavioral testing [OF—*F*_(4, 60)_ = 6.20; *p* < 0.001; Elevated Plus—*F*_(6, 74)_ = 6.63; *p* < 0.001] regardless of siRNA treatment (Figures 4, 5). Prior stress had a significant main effect corticosterone levels [*F*_(1, 74)_= 14.91; *p* < 0.001] but RNAi treatment alone did not [*F*_(1, 74)_ = 0.90; *p* = 0.345]. There was a significant interaction of RNAi x Prior Stress [*F*_(1, 74)_ = 7.40; *p* = 0.008]. Highest concentrations of corticosterone were found after EPM testing in control rats that had been restrained the day before. Knocking down CRF in the CeA blocked this heightened corticosterone response (Figure 4; *p* < 0.05).

### Behavioral testing

#### Open field

**Figure 6:**
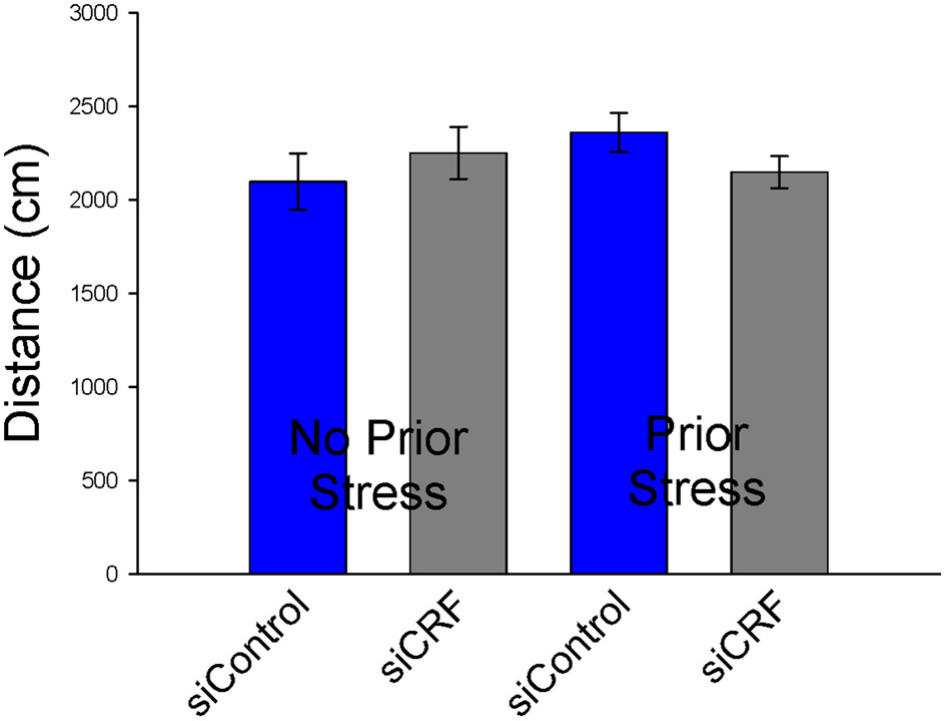
**Distance traveled (cm ± SEM) in Open Field was not significantly altered by siCRF treatment or restraint stress**.

**Figure 7:**
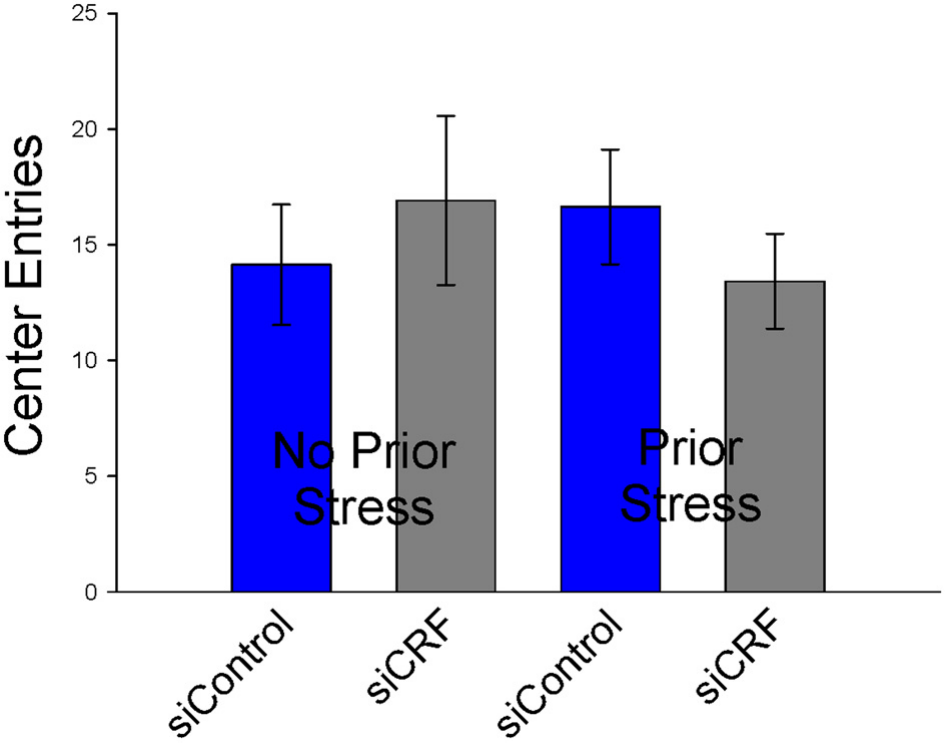
**Number of entries into center (± SEM), a measure of anxiolytic behavior, were not affected by siRNA treatment or restraint stress**.

Knockdown of CRF in the CeA had no effect on locomotion or measures of anxiety-like behavior in the OF. Total distance traveled was not different between treatment groups [Figure 6; *F*_(3,50)_ = 0.94; *p* = 0.43]. Measures of anxiety-like behavior included: frequency of entries into the center [Figure 7; *F*_(3,50)_ = 0.41; *p* = 0.75], duration of time spent in center [*F*_(3,50)_ = 0.36; *p* = 0.78] and rearing [*F*_(3,50)_ = 2.17; *p* = 0.10].

#### Elevated plus maze

**Figure 8:**
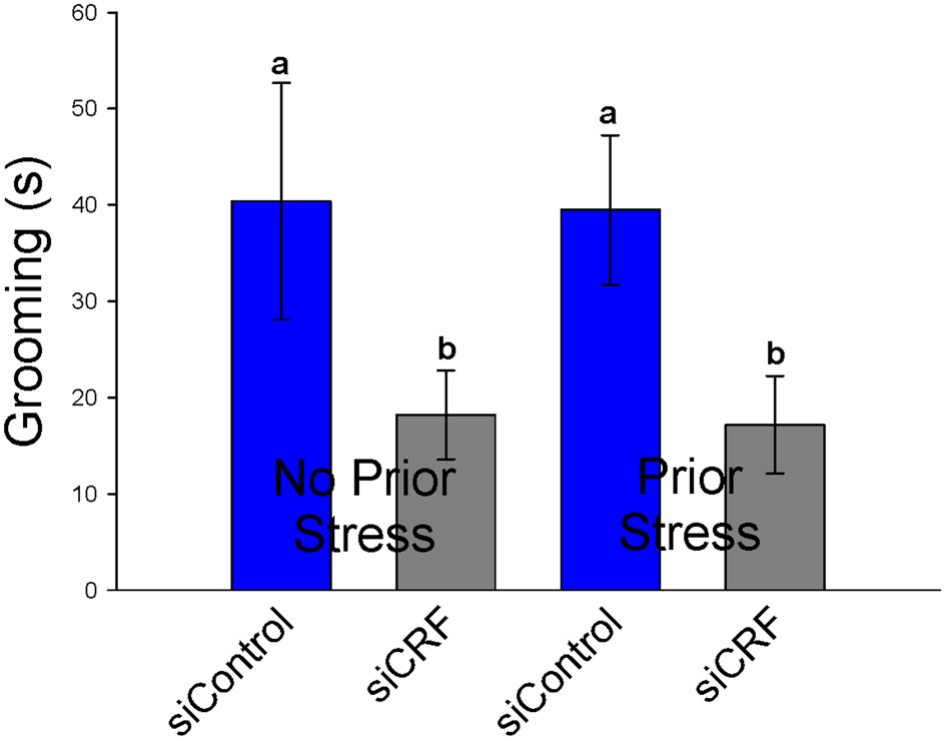
**Grooming Behavior on the EPM (s ± SEM) was significantly attenuated in rats with decreased CRF in CeA (*p* < 0.05).** Prior restraint stress had no effect on this behavior.

Standard measures of anxiety-like behavior on the EPM (% time spent and % entries on open vs. closed arms) were not altered by siRNA treatment [*F*_(3, 41)_ = 1.23; *p* = 0.311 and *F*_(3, 41)_= 0.98; *p* = 0.414, respectively]. Interestingly, frequency of open entries was significantly increased by knocking down CRF expression in the CeA [*F*_(3, 41)_ = 3.05; *p* < 0.05]. This measure does not appear to be caused by increased locomotion as total distance traveled was not different significantly between groups [*F*_(3, 41)_ = 1.65; *p* = 0.20]. Time spent grooming was also significantly attenuated by siRNA treatment regardless of prior stress [*F*_(3, 41)_ = 3.05; *p* < 0.05]. Rats that received CRF siRNA (siCRF) groomed less than rats receiving siControl (Figure 8). Prior stress had no effect on this parameter (40.4 s ± 12.3 and 39.5 ± 7.7). Rearing [*F*_(3, 41)_ = 1.669; *p* = 0.19] and number of head peeks [*F*_(3, 41)_ = 0.512; *p* = 0.68] were not affected by treatment.

## Discussion

We sought to determine if CRF in the CeA plays a role in stress-induced anxiety-like behaviors and HPA activation by using RNAi techniques to locally and rapidly (within 2 days) silence CRF expression in the CeA. This study demonstrates that while CRF in the CeA plays a role in the expression of certain CRF-related behaviors and HPA responses, knocking down amygdalar CRF expression is not sufficient to block the expression of anxiety-like behaviors on either the EPM or in the OF. Grooming, a CRF-inducible behavior, was attenuated in siCRF treated animals while measures of HPA activation (plasma corticosterone) were decreased by siCRF treatment animals with a prior stress exposure.

Prior exposure to a stressor sensitized an amygdalar CRF effect on stress-induced HPA activation. In these stress-challenged animals silencing CRF in the CeA significantly attenuated corticosterone responses to a behavioral stressor. Corticosterone release induced by restraint stress was not affected by treatment but was significantly attenuated in siCRF animals in response to behavioral anxiogenic stimuli 24 h after restraint. Thus, it appears that the role of CRF in the CeA in mediating stress-induced HPA activation is context specific and demonstrates plasticity in response to prior stress history. Activation of the CeA has been shown to activate HPA axis (Herman et al., 2005). One possibility is an indirect CRF activation of the BNST. CRF neurons in the paraventricular nucleus receive input from the CeA both directly and indirectly through the BNST among other regions (Gray et al., 1989; Cullinan et al., 1993; Gray, 1993). However, direct input from the CeA is minor compared to that of the BNST. This anatomical evidence led to the proposal that the CeA influences the HPA axis via complex multisynaptic pathways that likely include the BNST (Herman et al., 1994, 2005; Prewitt and Herman, 1998). Interestingly, chronic overexpression of CRF in the CeA (10 weeks) leads to increased expression of both CRF and AVP in the PVN which correlated with increased measures of HPA hyperactivity (Flandreau et al., 2012).

Restraint stress has been demonstrated to cause activation and enhancement of CRF-mediated responses. It causes a rapid increase in both CRF mRNA and peptide in neurons of the CeA (Mamalaki et al., 1992; Kalin et al., 1994; Hsu et al., 1998; Makino et al., 1999) and directly stimulates CRF release in the CeA (Merlo-Pich et al., 1995). Prior restraint stress also causes a suppression of exploratory behavior in a number of paradigms that can be attenuated by CRF receptor antagonists (Berridge and Dunn, 1987, 1989). Restraint stress increases CRF-enhanced acoustic startle (Pelton et al., 1997) and markedly enhances CRF induced firing of populations of dorsal raphe serotonergic neurons (Lowry et al., 2000). This sensitization of CRF responses by prior stressors has also been demonstrated for CRF-mediated signaling specifically in the CeA. Administration of CRF (icv) alone has no effect on serotonin signaling in the CeA, however, following restraint (24 h prior) CRF significantly attenuated serotonin release as measured by microdialysis and hplc in freely moving rats (Ronan et al., 1999). In this experiment prior stress exposure unmasked a role for CeA CRF knockdown in stress-induced activation of the HPA and anxiety-like responses.

Another behavioral variable that is reliably increased by icv CRF administration is locomotion (Dunn and Berridge, 1990; Koob, 1999; Lowry et al., 1996). In our study, decreases in CeA CRF did not significantly alter locomotion either in the OF or on the EPM. In agreement with our findings two other studies found no effect of long-term viral mediated amygdalar CRF over- or underexpression on locomotion in homecage environments (Regev et al., 2011, 2012). Other studies, however, have suggested an inhibitory role of amygdalar CRF on locomotion. Rats treated with corticosterone early in development had decreased numbers of CRF-immunopositive neurons in the CeA and this correlated with increased locomotion (Roskoden et al., 2005). It is difficult to ascribe a central role of CRF to this altered locomotion since there are certainly many other systems that could contribute that would have been altered by early exposure to corticosterone. More recently, specific viral-mediated overexpression of CRF in the amygdala also led to decreased locomotion in the OF but not other tests leading the authors to postulate that amygdalar CRF does not cause a locomotor deficit but rather a “psychomotor retardation in a novel environment” (Flandreau et al., 2012). Together with our findings the evidence points to no significant role of amygdalar CRF on baseline locomotor activity.

Our results only partially support the literature suggesting a central role for CRF in the CeA with the expression of anxiety-like behavior and the hypothesized central role of CRF in stress-induced anxiety disorders (Davidson, 2002; Schwartz et al., 2003; Ronan and Summers, 2011). Inhibiting expression of CRF in the CeA had no effect on standard measures of anxiety-like behaviors on the EPM (% time or % entries on the open vs. closed arms) or in the OF (% time in center). In this experiment, grooming behavior on the EPM was significantly attenuated by loss of CRF in CeA regardless of whether or not animals had been previously exposed to restraint stress. Grooming behavior on the EPM is often used as a corollary measure of anxiety-like behavior (Bolanos et al., 2003; Estanislau, 2012; Tapia-Osorio et al., 2013), however, this is not universally accepted (Bolles, 1960; van Erp et al., 1994). Self-grooming is a behavioral response in rodents to stressful or anxiogenic stimuli (Spruijt et al., 1992). Central administration of CRF (icv) has long been known to cause increased grooming behavior (Dunn et al., 1987). This study highlights the important role of amygdalar CRF in this behavior. The significance, if any, of the finding that on the EPM, siCRF treatment rats had significantly more open entries than controls is unclear. When normalized to number of closed entries this significance is lost. Perhaps it is simply an expression of increased motor activity, though total locomotion did not differ. Or it could be indicative of an increase in exploratory behavior. Thus, knocking down CRF in the CeA alters some stress-induced behaviors it is not sufficient to completely attenuate anxiety-like responses.

Even though there is a wealth of evidence suggesting an anxiogenic role for CRF and the amygdala it is likely that CRF-mediated anxiety-like behaviors, like that hypothesized for endocrine responses, are mediated in part by other brain regions as well. Studies in rats, cats, and rabbits have shown that electrical stimulation of the CeA causes anxiety-like responses (Kaada et al., 1954; Hilton and Zbrozyna, 1963; Heinemann et al., 1973; Mogenson and Calaresu, 1973) and conversely, lesions of the CeA attenuate anxiety-like responses (Moller et al., 1997; Kalin et al., 2004). The primate amygdala plays a role in certain anxiety-like behaviors such as anxiety-related defensive responses, which may have a conditioned fear component (Kalin et al., 2004). The amygdala has been implicated in anxiety responses in a novel environment such as the EPM. However, amygdala lesions do not affect anxiogenic responses on the EPM except in rats that had received restraint stress (Moller et al., 1997).

It has been suggested that the CeA may be more specific to conditioned anxiety or fear, whereas the BNST may be more involved in unconditioned anxiogenic effects (Walker and Davis, 1997; Davis and Shi, 1999; Davis et al., 2010). The BNST and the CeA are anatomically, neurochemically, cytoarchitecturally, and embryologically related (Alheid and Heimer, 1988). The BNST is considered a site of convergence of information from brain regions associated with the control of emotional, cognitive, autonomic and behavioral responses related to stress. (Alheid and Heimer, 1988; Casada and Dafny, 1993a,b; Ciriello and Janssen, 1993; Cullinan et al., 1993). The lateral subdivision of the anterior BNST, containing high concentrations of CRF neurons is extensively connected with several brain areas that coordinate autonomic, neuroendocrine and behavioral responses to stress, such as a reciprocal connection with the CeA, parabrachial nucleus and PVN (De Olmos and Ingram, 1972; Moga et al., 1989; Moga and Saper, 1994; Dong et al., 2001; Dong and Swanson, 2004).

The involvement of CRF systems in both the BNST and CeA has been demonstrated for other stress-induced behaviors. CRF systems in both the CeA and BNST appear to be involved in stress-induced relapse to cocaine seeking behavior in rats. CRF microinfusions into the BNST but not the CeA mimic the effects of footshock on reinstatement (Erb et al., 2001). Also, infusions of the CRF_1_ antagonist D-Phe CRF_12−41_ into the CeA are ineffective at blocking reinstatement but doses 10-fold lower in the BNST are capable of blocking the behavior. Lesions of the CeA lead to an attenuated reinstatement but do not block the behavior completely. Together these findings suggest a role for the CRF-containing pathway from the CeA to the BNST in stress-induced reinstatement but CRF release in the BNST appears to be the critical factor controlling this behavior. This release could be both from fibers originating in the CeA as well as from local circuits in the BNST. Another behavior mediated by CRF systems in both the CeA and BNST is fear-potentiated acoustic startle. The acoustic startle reflex is an unconditioned response to a loud noise. The startle reflex that is elicited by noise in a non-paired context can be increased in amplitude if the startle noise is given in the presence of a cue that has been previously paired with shock (Brown et al., 1951; Davis and Astrachan, 1978; Davis, 1990; Yeomans and Pollard, 1993). Electrolytic lesions of either the amygdala (Hitchcock and Davis, 1986) or the amygdalofugal pathways (Hitchcock and Davis, 1987) can block this fear potentiated startle but not the startle response itself. The direct pathway from the amygdala to the nucleus reticularis pontis caudalis was originally proposed as the critical pathway mediating fear-potentiated startle (Hitchcock and Davis, 1991). Further studies have demonstrated that this pathway is not direct. It is relayed by a synapse in the rostral midbrain (Yeomans and Pollard, 1993; Zhao and Davis, 2004). Injections of CRF (icv) can also enhance acoustic startle response in a non-paired context. This effect is mediated by the BNST. Lesions of the BNST but not CeA block CRF-mediated potentiation of acoustic startle. Also, CRF microinjected into BNST but not CeA mimics the effects of icv CRF and CRF antagonists in the BNST block CRF enhanced startle (Lee and Davis, 1997). Though the CeA is critical to the expression of fear-potentiated startle it is not critical to the expression of CRF enhanced startle. Much like the story with stress-induced relapse, it appears that the effects of CRF are mediated in the BNST, at least in part by a CRF pathway from the CeA. Thus, it is likely that activation of the BNST may be a critical output for behavioral responses.

In conclusion, CRF in the CeA certainly contributes to the expression of endocrine responses to stressors but has much less effect on behavioral responses. Silencing CRF expression in the amygdala attenuates a heightened HPA activation in response to a behavioral stressor in animals with prior stress exposure. Unconditioned stress-induced anxiety-like behavioral responses are attenuated by loss of CRF in the CeA in animals regardless of stress history. However, knocking down expression of CRF in the CeA is not sufficient to completely block expression of anxiety-like responses suggesting that amygdalar CRF is part of a wider response coordinated by this and other brain regions/neurotransmitter systems. This study adds further weight to the body of evidence linking CRF signaling to stress-induced anxiety disorders and helps to clarify the specific contribution of amygdalar CRF to stress responsiveness.

### Conflict of interest statement

The authors declare that the research was conducted in the absence of any commercial or financial relationships that could be construed as a potential conflict of interest.
